# Supplementary material for: Circulating Metabolites as Biomarkers of Disease in Patients with Mesial Temporal Lobe Epilepsy
Source: Metabolites. 2022 May 17;12(5):446. doi: 10.3390/metabo12050446 (PMC9148034; doi:10.3390/metabo12050446)
Supplement: Supplementary file 1 [file metabolites-12-00446-s001.zip › Supplementary file 2.pdf]

Metabolomic Data Analysis with MetaboAnalyst 5.0

Name: guest5723476134630056340

March 15, 2022

1 Background

Metabolite Set Enrichment Analysis (MSEA) is an approach used to identify biologically meaningful patterns significantly enriched in quantitative metabolomics data. In conventional approaches, metabolites are evaluated individually for their significance under the conditions of the study. Next, the compounds that have passed a certain significance level are combined to see if any meaningful pattern can be found. In contrast, MSEA directly investigates a set of functionally related metabolites without the need to preselect compounds based on some arbitrary cut-off threshold. In addition, MSEA contains a collection of metabolites set libraries and the implementation of user-friendly web interfaces. As a result, it has the potential to identify subtle but significant changes among a group of related compounds, which may go undetected with the conventionalapproaches. MSEA is a metabolomic version of the popular Gene Set Enrichment Analysis (GSEA) software. GSEA is widely used in genomics data analysis and has proven to be a powerful alternative to conventional approaches.

2 MSEA Overview

MSEA consists of four steps - data input, data processing, data analysis,and results download. Different analysis procedures are performed based on different input types. In addition, users can browse and search the metabolite set libraries and upload their self-definedmetabolite sets for enrichment analysis. Finally, users can also map metabolite names among compound names, synonyms, and major database identifiers.

3 Data Input

MSEA offers three enrichment analysis algorithms. Accordingly, three different types of data inputs are required by these three approaches:

A list of compound names - entered as a one-column data (*Over Representation Analysis (ORA)*);

A single measured biofluid (urine, blood, CSF) sample- entered as tab-separated two-column data with the first column for compound name, and the second for concentration values (*Single Sample ProFIling (SSP)*);

A compound concentration table - entered as a comma-separated (.csv) file with each sample per row and each metabolite concentration per column. The first column is sample names, and the second column is for sample phenotype labels (*Quantitative Enrichment Analysis (QEA)*). For this type of analysis, users should select "Over Representation Analysis" (ORA), which requires a list of compound names as input.

4 Data Processing

The first step is to standardize the compound labels. It is an essential step since the compound labels will subsequently be compared with compounds in the metabolite set library. MSEA has a built-in tool to convert common compound names, synonyms, identifiers used in HMDB ID, PubChem, ChEBI, BiGG, METLIN, KEGG, or Reactome. **Table S3** shows the conversion results. Note:*1* indicates an exact match, *2* indicates an approximate match, and *0* indicates no match. A text file containing the results can be found in the downloaded file *name map.csv*

Table S3: Result from Compound Name Mapping

	Query	Match	HMDB	PubChem	KEGG	SMILES	Comment
1	Lactate	L-Lactic acid	HMDB0000190	61503	C00186	C[C@@H](C(=O)O)O	1
2	Glucose	D-Glucose	HMDB0000122	5793	C00221	C([C@@H]1[C@H]([C@@H]([C@H](C(O1)O)O)O)O)O	1
3	Isoleucine	L-Isoleucine	HMDB0000172	6306	C00407	CC[C@H](C)[C@@H](C(=O)O)N	1
4	Proline	L-Proline	HMDB0000162	145742	C00148	C1C[C@H](NC1)C(=O)O	1

The second step is to check concentration values. The concentration must be measured in "*umol"* for blood and CSF samples, thus enabling SSP analysis. The urinary concentrations must be first converted to *umol/mmol creatinine* to compare with reported concentrations in the literature. No missing or negative values are allowed in SSP analysis. The concentration data for QEA analysis is more flexible. Users can upload either the original concentration data or normalized data. Missing or negative values are allowed (coded as *NA*) for QEA.

5 Selection of Metabolite Set Library

Before proceeding to enrichment analysis, a metabolite set library has to be chosen. MSEA offers seven built-in libraries:

Metabolic pathway associated metabolite sets (*currently contains 99 entries*);

Disease-associated metabolite sets (reported in the blood) (*currently contains 344 entries*);

Disease-associated metabolite sets (reported in urine) (*currently contains 384 entries*)

Disease-associated metabolite sets (reported in the CSF) (*currently contains 166 entries*)

Metabolite sets associated with SNPs (*currently contains 4598 entries*)

Predicted metabolite sets based on computational enzyme knockout model (*currently contains 912 entries*)

Metabolite sets based on locations (*currently contains 73 entries*)

Drug pathway associated metabolite sets (*currently contains 461 entries*)

In addition, MSEA also allows user-defined metabolite sets to be uploaded to perform enrichment analysis on arbitrary groups of compounds. The metabolite set library is a two-column comma-separated text file with the first column for metabolite set names and the second column for its compound names (**must use HMDB compound name**) separated by "; ". Please note that the built-in libraries are mainly from human studies. Therefore, users must upload their self-defined metabolite set libraries for enrichment analysis using data from other organisms.

6 Enrichment Analysis

Over Representation Analysis (ORA) is performed when a list of compound names is provided. The list of compounds can be obtained by conventional feature selection methods, a clustering algorithm, or compounds with abnormal concentrations detected in SSP to investigate if some biologically meaningful patterns can be identified.

ORA was implemented using the *hypergeometric test* to evaluate whether a specific metabolite set is present more than expected by chance compared to a specific list. Also, one-tailed p-values, corrected for multiple comparisons, are provided. **Figure S2 and Table S4** summarize the results.

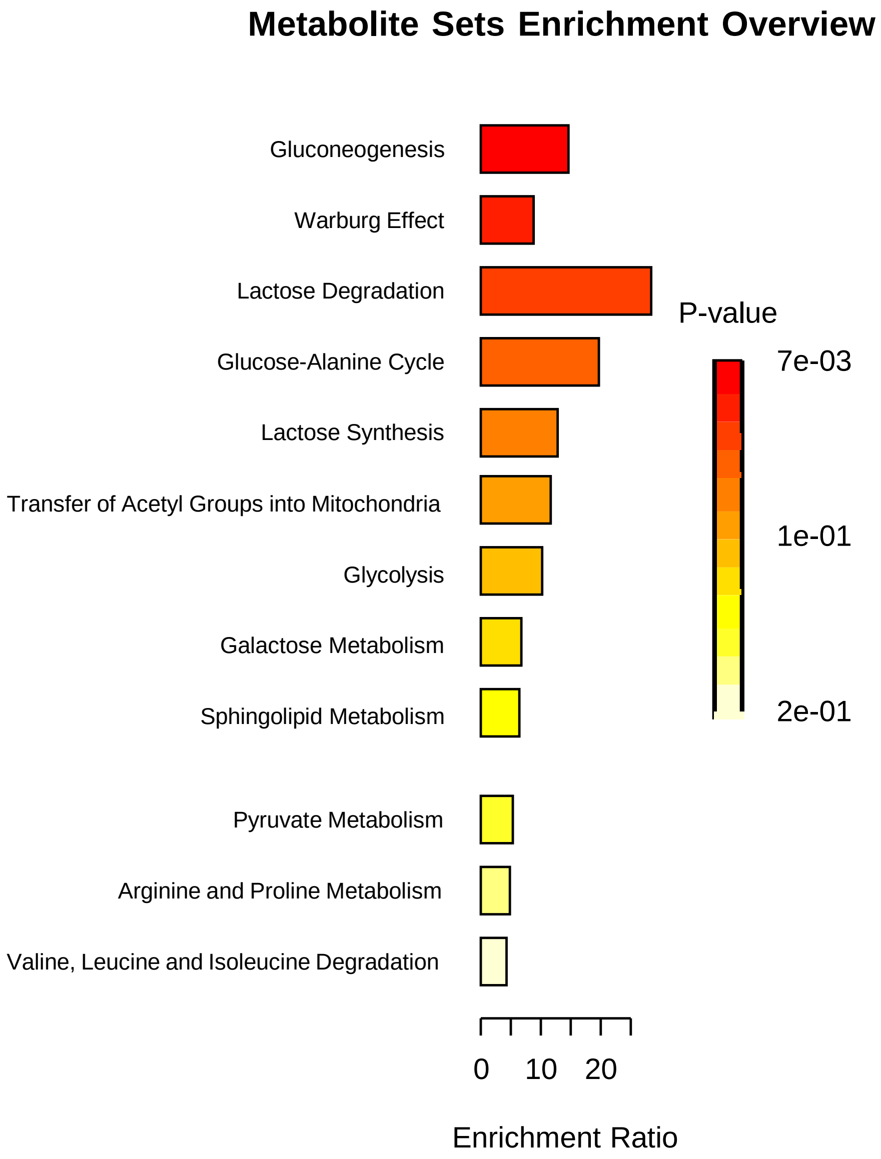

Figure S2: Summary Plot for Over Representation Analysis (ORA)

Table S4: Result from Over Representation Analysis

	total	expected	hits	Raw p	Holm p	FDR
Gluconeogenesis	35	0.14	2	6.53E-03	6.40E-01	6.40E-01
Warburg Effect	58	0.23	2	1.76E-02	1.00E+00	8.61E-01
Lactose Degradation	9	0.04	1	3.47E-02	1.00E+00	1.00E+00
Glucose-Alanine Cycle	13	0.05	1	4.99E-02	1.00E+00	1.00E+00
Lactose Synthesis	20	0.08	1	7.60E-02	1.00E+00	1.00E+00
Transfer of Acetyl Groups into Mitochondria	22	0.09	1	8.33E-02	1.00E+00	1.00E+00
Glycolysis	25	0.10	1	9.43E-02	1.00E+00	1.00E+00
Galactose Metabolism	38	0.15	1	1.41E-01	1.00E+00	1.00E+00
Sphingolipid Metabolism	40	0.16	1	1.48E-01	1.00E+00	1.00E+00
Pyruvate Metabolism	48	0.19	1	1.75E-01	1.00E+00	1.00E+00
Arginine and Proline Metabolism	53	0.21	1	1.92E-01	1.00E+00	1.00E+00
Valine, Leucine and Isoleucine Degradation	60	0.23	1	2.15E-01	1.00E+00	1.00E+00

7 R Command History

- "mSet<-InitDataObjects(\"conc\", \"msetora\", FALSE)"
- "cmpd.vec<-c(\"Lactate\",\"Glucose\",\"Isoleucine\",\"Proline\")"
- "mSet<-Setup.MapData(mSet, cmpd.vec);"
- "mSet<-CrossReferencing(mSet, \"name\");"
- "mSet<-CreateMappingResultTable(mSet)"
- "mSet<-SetMetabolomeFilter(mSet, F);"
- "mSet<-SetCurrentMsetLib(mSet, \"smpdb_pathway\", 2);"
- "mSet<-CalculateHyperScore(mSet)"
- "mSet<-PlotORA(mSet, \"ora_0_\", \"net\", \"png\", 72, width=NA)"
- "mSet<-PlotEnrichDotPlot(mSet, \"ora\", \"ora_dot_0_\", \"png\", 72, width=NA)"
- "mSet<-CalculateHyperScore(mSet)"
- "mSet<-PlotORA(mSet, \"ora_1_\", \"net\", \"png\", 72, width=NA)"
- "mSet<-PlotEnrichDotPlot(mSet, \"ora\", \"ora_dot_1_\", \"png\", 72, width=NA)"
- "mSet<-SaveTransformedData(mSet)"

[15] "mSet<-PreparePDFReport(mSet, \"guest5723476134630056340\")\n"

————————————

The report was generated on Tue, March 15, 19:11:15 2022 with R version 4.0.2 (2020-06-22).
